# Mucocèle sphénoïdale révélée par une exophtalmie

**DOI:** 10.11604/pamj.2015.22.227.8107

**Published:** 2015-11-11

**Authors:** Rim Lahiani, Madiha Mahfoudhi

**Affiliations:** 1 Service ORL, Hôpital Charles Nicolle, Tunis, Tunisie; 2 Service de Médecine Interne A, Hôpital Charles Nicolle, Tunis, Tunisie

**Keywords:** Mucocèle, sinus sphénoïdal, exophtalmie, Mucocele, sphenoid sinus, exophtalmia

## Image en medicine

Une mucocèle est une tumeur bénigne pseudo-kystique, se développant dans les sinus de la face. Elle siège le plus souvent au niveau du complexe éthmoïdo-frontal. La localisation sphénoïdale est rare (1 à 2 % des mucocèles sinusiennes). La symptomatologie non spécifique des mucocèles sphénoïdales doit impérativement conduire à la réalisation d'une endoscopie nasale et la demande d'une imagerie afin de proposer une prise en charge thérapeutique précoce évitant ainsi les séquelles ophtalmologiques irréversibles. Homme âgé de 45 ans sans antécédents particuliers a consulté pour exophtalmie, baisse de l'acuité visuelle et épistaxis. L'examen physique a objectivé une exophtalmie droite et une baisse de l'acuité visuelle. L'examen biologique a retrouvé un discret syndrome inflammatoire. L'endoscopie nasale a révélé un bombement du récessus ethmoïdo-sphénoïdal. La TDM du massif facial en coupe axiale a montré un processus expansif sphénoïdal et ethmoïdal postérieur droit qui comprime le nerf optique et souffle les parois osseuses. L'IRM du massif facial en coupes axiales et en pondération T1 injecté et T2 a objectivé le même processus en hypersignal après injection de gadolinium et en hypersignal T2. Plusieurs diagnostics ont été évoqués en particulier un lymphome ou une néoplasie solide. Le traitement s'est basé sur une marsupialisation et une sphénoïdotomie. Les suites opératoires étaient simples. L'examen anatomo-pathologique a conclut à une mucocèle. L'évolution était marquée par une régression incomplète des troubles visuels sans notion de récidive avec un recul de 36 mois.

**Figure 1:**
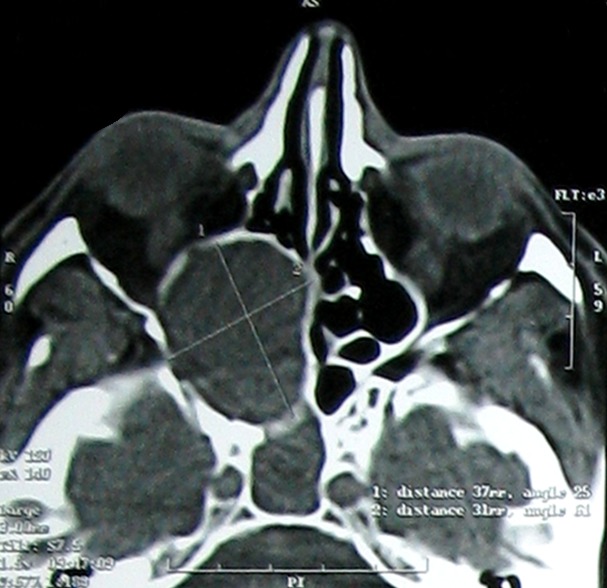
TDM du massif facial en coupe axiale: processus expansif sphénoïdal et ethmoïdal postérieur droit qui comprime le nerf optique et souffle les parois osseuses

